# Evaluation of the Nijmegen Cochlear Implant Questionnaire in Danish

**DOI:** 10.1055/s-0044-1788598

**Published:** 2025-01-22

**Authors:** Charlotte Skov Neumann, Jesper Hvass Schmidt

**Affiliations:** 1 Research Unit for ORL – Head and Neck Surgery and Audiology, Odense University Hospital, Odense, Denmark; 2 Department of Clinical Research, University of Southern Denmark, Odense, Denmark; 3 OPEN, Odense Patient Data Explorative Network, Odense University Hospital, Odense, Denmark

**Keywords:** cochlear implant, Nijmegen cochlear implant questionnaire, quality of life, cochlear implant surgery, DA-NCIQ

## Abstract

**Introduction** The Nijmegen cochlear implant questionnaire (NCIQ) is a quantifiable self-assessment health-related quality of life (HRQoL) tool used internationally to determine quality of life (QoL) in cochlear implant (CI) users and to evaluate the implant's subjective benefits.

**Objective** This study aimed to validate the Danish version of the questionnaire (DA-NCIQ) with a test–retest including 60 participants (30 CI users and 30 CI candidates).

**Methods** The intraclass correlation coefficients (ICC) were calculated to evaluate the temporal stability of the participants' answers and the internal consistency of the questionnaire domains was determined using the Cronbach alpha in order to compare these results with the NCIQ's other language versions.

**Results** The DA-NCIQ was found to have Cronbach alpha coefficients between 0.7 and 0.91, as well as test–retest reliability with ICC values between 0.7 and 0.92. These findings were similar to the original and other language versions of this questionnaire. The Cronbach alpha coefficients varied between 0.73 and 0.89, while the ICC test–retest reliability varied between 0.64 and 0.85. Furthermore, the present study found that participants with CIs had an improved HRQoL in all subdomains, except for the advanced sound perception one, when compared to the CI candidates.

**Conclusion** The results supported the DA-NCIQ as a reliable instrument to measure the subjective benefits of CIs in postlingually deafened/hearing-impaired adults.

## Introduction

Regardless of the age at which disabling hearing impairment develops, it is known to have damaging consequences for interpersonal communication, psychosocial wellbeing, quality of life (QoL), and economic independence. 1 Studies have shown that cochlear implants (CIs) help the hearing-impaired to improve not only their hearing, speech perception, and communicative functions, but also their QoL. 2 3 4 5

The evaluation of CI candidates in Denmark consists of an interdisciplinary evaluation of the patients' auditive, linguistic, and cognitive functions, and it is an important factor that the patient is able to, and motivated for, rehabilitation after the surgery. 6 7

Even though general health in CI users is known to be the same as for normal-hearing individuals, they still face some challenges concerning their hearing abilities after surgery. 8

Health-related QoL (HRQoL) questionnaires can be used to determine the subjective benefits of a CI, and one of the main goals of this treatment is to improve patients' HRQoL. It is relevant to have a validated and standardized instrument to measure the HRQoL in CI users, as well as to monitor how the patients improve following surgery.

However, outcome measures used to assess the benefit of a CI often focus on word and sentence recognition in a fixed setting, while the individual hearing experiences and ability to communicate in various types of settings are far more complex than the typical test measurements can assess. 9 10 11 12

The Nijmegen cochlear implant questionnaire (NCIQ) was originally developed in the Netherlands and is designed to measure the HRQoL in adult CI users. It is a questionnaire containing 60 questions, which can be grouped into 3 domains: physical, psychological, and social functioning. These domains consist of 6 subdomains: basic sound perceptions, advanced sound perception, speech production, self-esteem, activity, and social interaction. Each subdomain consists of 10 items, formulated as statements with five possible answers on a 5-point Likert-type scale going from ‘never’ to ‘always’ (55 statements), or from ‘no’ to ‘quite well’ (5 statements). The patient is asked to choose the answer that fits best with their experience in relation to each question. If a statement does not apply to a patient, a sixth answer: ‘not applicable’, can be used. 13

The original version of NCIQ was found to be reliable in its psychometric characteristics. It has been translated into and validated in other languages like Turkish, Italian, Spanish, Chinese, and Brazilian Portuguese, 14 15 16 17 18 and is already being used by researchers as a measure of HRQoL in CI users. 19 20

This questionnaire was recently translated into Danish, but the evaluation of the comparability with the original and other translations is still missing. Therefore, the aim of this study was to verify the internal consistency, and the test–retest reliability of the Danish version of the NCIQ (DA-NCIQ, Supplemental file 1 ) and to investigate the scores of Danish CI candidates and users.

## Methods

### The Translation

The NCIQ was recently translated from English into Danish. Cross-cultural adaptions were made using the practice guide for translating and adapting hearing-related questionnaires for different languages and cultures, provided by Hall et al. 21

Step 1: First, thorough research was done to ensure no other Danish version of the NCIQ existed. The characteristics of the target population and administration elements were considered and discussed.
Step 2: A native English-speaking consultant with Danish as a second language performed the translation. The consultant was briefed on the instrument, the clinical concepts underlying the health condition of interest, and the considerations made in step 1. Danish audiologists experienced with CIs from the Eastern Danish CI center then processed the translation, focusing on the correct use of language, and was performed to ensure the translation was semantically identical to the English version.
Step 3: The Danish version was then translated back into the source language and the “back translation” was reviewed against the English version of the NCIQ. The Danish translation was evaluated and modified continually throughout this process.
Step 4: The translations were reviewed by the translator and clinicians to reach cross-cultural equivalence between the original and Danish versions.
Step 5: A small pilot study was performed involving three hearing-impaired patients to ensure the questions were culturally appropriate.
Step 6: The results of the field-testing were highlighted and reviewed. Significant and final modifications were made. The translation underwent thorough proofreading, and a written report of the process was made. The report is available upon request.

### Recruitment and Study Population

A total of 60 adult participants were recruited at the Department of Audiology, Odense University Hospital (OUH) in the period of September 2019 through July 2020. The participants were divided into two groups. The first group consisted of 30 (17 females, mean age: 60 years, SD: 15 years) stable CI users with a minimum 1 year of experience, with limited expected improvement of CI outcome (post-CI). The participants in this group would be unilateral, bilateral, and bimodal (CI on one ear, hearing aid on the other ear) users. The participants were recruited during a follow-up visit at the Department of Audiology of the OUH. The second group was composed of 30 (15 females, mean age: 66 years, SD 11 years) participants referred for CI candidacy evaluation and therefore waiting to get their first CI (pre-CI) ( Table 1 ). Only participants who fulfilled the CI candidacy criteria were included in the pre-CI group. Both groups were expected to read and understand Danish on a sufficient level. A total of 63 participants met the inclusion criteria, of whom 3 were excluded due to their inability to read and understand the DA-NCIQ.

**Table 1:** Demographic and clinical characteristics of the post-CI (experience > 1 year) and pre-CI group, who were evaluated for implant candidacy

Demographics characteristics	Pre-CI (n = 30)	Post-CI (n = 30)
Sex (males/females)	15/15	13/17
Age (mean years ± SD)	66 ± 11 (range 43–82)	60 ± 15 (range 25–80)
Age at onset of hearing impairment (mean years ± SD)	45 ± 23 (range 2–70)	27 ± 20 (range 2–60)
Age at CI surgery (mean years ± SD)	−	55 ± 17 years (range 14–78)
Unilateral CI users (n)	−	18
Bilateral CI users (n)	−	12
Educational level		
Lower	8	10
Secondary	16	17
University	6	3
Paid employment		
Yes	12	11
No	18	19
Living situation:		
Alone	5	10
With others	25	20
Cause of deafness/hearing impairment:		
Unknown	8	5
Presbycusis	1	−
Hereditary	10	9
Meningitis	−	4
Noise damage	−	2
Sudden deafness	4	2
Meniérès disease	1	1
Trauma	−	1
Vascular	1	2
Otosclerosis	3	−
Rhesus immunization	1	−
Infectious disease in fetal life	1	4

**Abbreviation:** CI, cochlear implant; SD, standard deviation.

The most frequent cause of deafness/hearing impairment in both the pre- and post-CI groups was hereditary hearing loss, while the second most frequent cause was of unknown etiology ( Table 1 ).

The most frequent, highest educational level was secondary school in both groups.

Due to the average high age, more participants were labeled ‘unemployed’ because of their retirement status.

The largest difference between the two groups were in the age at onset of hearing impairment/deafness, which were 27 ± 20 years in the post-CI group and 45 ± 23 years in the pre-CI group.

The patient's mean age, as well as mean age at hearing impairment/deafness onset, were lower in the post-CI group. The mean age at implantation in the post-CI group was 55 ± 17 years (range 14–78 years).

### Procedure

The participants received oral and written information about the project and were asked to sign a written consent form to enter the study. They were then asked to answer the DA-NCIQ on the day of their clinical visit and to provide information about the underlying cause of their hearing impairment, age at the time of diagnosis of the hearing impairment, current use of hearing aid/CI, if they are living alone or with others, educational level and occupational status ( Table 1 ).

The DA-NCIQ was administered twice in a test-retest design. The patients who had filled out the first questionnaire at the Department of Audiology during the clinical visit (test), filled it again via their personal electronic postal box (secure email) 2 weeks later (retest). The interval period of 2 weeks was chosen because no considerable change in hearing and benefit from the current hearing aid solution was expected to happen within this period. The participants couldn't look at their previously answered questionnaire.

The speech identification scores (SIS) of 25 monosyllabic Danish words, presented in quiet and free field at 65 dB sound pressure level (SPL), were collected from the patients in the best aided condition with new and recently fitted hearing aids. These scores included purely auditory scores without visual lip-reading cues from a TV screen (auditory SIS) as well as audio-visual scores consisting of audio with visual lipreading cues from the TV screen (audio-visual SIS). The two test conditions were also performed in the presence of noise with a Signal to Noise Ratio (SNR) of 0 dB.

The participants with a SIS below 88% were considered to be hearing impaired, as normal hearing individuals are expected to achieve an Auditory SIS between 88 and 100% when tested in noise. 22

All data were stored in the research electronic data capture (REDCap) tool developed by Vanderbilt University, Nashville, Tennessee, United States, 23 24 and the REDCap database was hosted by the Odense Patient Explorative Network (OPEN) in the Region of Southern Denmark.

### Statistical Analyses

Statistical tests were performed using the STATA SE (Stata Corp., College Station, TX, USA) version 16.0.

Consistent with the approach outlined in the initial study by Hinderink et al., 13 missing values and responses marked as ‘not applicable’ were considered incomplete and thus excluded from the dataset. The response categories were Never = 1, Sometimes = 2, Regularly = 3, Usually = 4, Always = 5 and the response categories (1–5) were transformed, with values set as follows: 1 = 0; 2 = 25; 3 = 50; 4 = 75 and 5 = 100. For 27 questions the answer “Always” is the most negative answer, whereas the answer “Never” is the most positive answer. The 27 questions were therefore recoded and the scores were reversed. Thus, the values were set as follows for these 27 questions: 1 = 100, 2 = 75, 3 = 50, 4 = 25, 5 = 0.

The score of each subdomain was calculated as the sum of the scores of the 10 questions within the given subdomain, divided by the number of completed questions. If a participant had failed to answer a minimum of 7 out of 10 in a specific subdomain, the participant was excluded as described in Hinderick et al. 13

The internal consistency of each subdomain in the DA-NCIQ was assessed with the Cronbach alpha coefficient. Results between 0.7 and 0.95 were considered sufficient, whereas values below 0.7 and above 0.95 were suspected to offer limited evidence of internal consistency. 25

To evaluate the test–retest reliability of DA-NCIQ, estimates of intraclass correlation coefficients (ICC) and their 95% confidence intervals were calculated (based on the absolute-agreement, two-way mixed-effects model) by comparing the scores of each subdomain in the test and retest responses. The ICC values were interpreted as the following: less than 0.5 = poor reliability, between 0.5 and 0.75 = moderate reliability, between 0.75 and 0.9 = good reliability, and greater than 0.90 = excellent reliability. 26

## Results

Table 2 presents the speech identification scores (SIS) collected in the best aided condition in free field in quiet and in noise before surgery. The mean SIS in noise and in quiet was noticeably lower for the post-CI group compared to the pre-CI group. In both groups, the mean audiovisual score, where visual cues were used as well, were higher than the mean auditory score in both quiet and noise.

**Table 2:** Speech identification scores (SIS) in the best aided condition measured in free field (collected before CI surgery)

Free field hearing test	Pre-CI			Post-CI		
	Mean	SD	Range	Mean	SD	Range
Auditory SIS in quiet (%)	**52.6** (n = 28)	30.2	0–100	**30** (n = 16)	28.9	0–92
Audiovisual SIS in quiet (%)	**73** (n = 23)	21.8	8–100	**43** (n = 15)	29.3	0–92
Auditory SIS in noise (%)	**26.4** (n = 22)	25.2	0–88	**4** (n = 3)	6.7	0–12
Audiovisual SIS in noise (%)	**50.2** (n = 22)	22.6	0–96	**8** (n = 2)	11.3	0–16

**Abbreviation:** CI, cochlear implant; SD, standard deviation; SIS, speech identification scores.

Table 3 presents the scores achieved for each subdomain of the questionnaire ranging from 55.2 to 70.9 for the post-CI, and 44.8 to 67.8 for the pre-CI group. Both test and retest scores were higher for the post-CI group, except for the advanced sound perception subdomain. For both groups, scores from the test and retest questionnaires did not differ significantly from each other.

**Table 3:** Scores obtained by the 6 subdomains in the DA-NCIQ

Pre-CI test					Pre-CI retest				Mean (test + retest)
Subdomain	Mean	SD	Range	n	Mean	SD	Range	n	
Basic sound perception	46	18.7	17.5–82.5	30	43.5	18.8	17.5–86.1	30	**44.8**
Advanced sound perception	67.2	21.1	15.6–100	30	68.4	21.2	19.4–100	30	**67.8**
Speech production	50.1	14.4	25–87.5	30	49.9	16.0	22.5–88.9	30	**50.0**
Self-esteem	50.9	12.7	25–69.4	30	50.2	13.4	25–75	30	**50.6**
Activity	56.7	15.7	30.6–80	30	55	17.7	15–85.7	30	**55.9**
Social interactions	51.3	13.4	22.2–72.5	30	50.4	14.0	20–70	30	**50.9**
**Post-CI test**					**Post-CI retest**				
Basic sound perception	57.8	18.9	22.5–90	30	54.4	20.7	22.5–92.5	30	**56.1**
Advanced sound perception	66.7	16.8	30–100	30	66.8	16.5	32.5–97.5	30	**66.8**
Speech production	54.6	18.4	25–92.5	30	55.8	18.9	12.5–87.5	30	**55.2**
Self-esteem	57.6	12.8	20–75	30	59.6	14.2	27.5–82.5	30	**58.6**
Activity	69.6	15.8	30–97.5	30	72.2	14.7	36.1–100	30	**70.9**
Social interactions	62.9	11.6	36.1–84.4	30	65.2	10.7	42.5–86.1	30	**64.0**

**Abbreviation:** CI, cochlear implant; DA-NCIQ, Danish Nijmegen cochlear implant questionnaire; SD, standard deviation.

The internal consistency of the DA-NCIQ was assessed using the Cronbach alpha and is presented in Table 4 . The scores were within the range of 0.7 to 0.95 for all subdomains. The lowest scores were obtained by the social interaction (α = 0.79) subdomain in the post-CI group, as well as the speech production (α = 0.79), and the social interaction (α = 0.79) subdomains in the pre-CI group.

**Table 4:** Internal consistency of the subdomains in the DA-NCIQ using the Cronbach alpha

Subdomain	Test pre-CI	Retest pre-CI	Mean	Test post-CI	Retest post-CI	Mean
Basic sound perception	0.89	0.89	**0.89**	0.88	0.92	**0.90**
Advanced sound perception	0.89	0.92	**0.91**	0.83	0.84	**0.84**
Speech production	0.77	0.80	**0.79**	0.85	0.88	**0.87**
Self-esteem	0.82	0.81	**0.82**	0.77	0.82	**0.80**
Activity limitations	0.82	0.87	**0.85**	0.86	0.83	**0.85**
Social interactions	0.85	0.72	**0.79**	0.77	0.62	**0.70**

**Abbreviation:** CI, cochlear implant; DA-NCIQ, Danish Nijmegen cochlear implant questionnaire.

The test–retest reliability for each subdomain was estimated using the ICC, all presented in Table 5 . All subdomains had ICC values within 0.77 to 0.92, suggesting good or excellent reliability, except for the self-esteem and social interaction subdomains in the post-CI group and the self-esteem subdomain in the pre-CI group, all of which were within 0.7 to 0.73 and showed moderate reliability.

**Table 5:** Test–retest reliability calculated using the intraclass correlation coefficient (ICC)

	Pre-CI		Post-CI	
	ICC	95%CI	ICC	95%CI
Basic sound perception	**0.89**	0.79–0.94	**0.83**	0.69–0.92
Advanced sound perception	**0.92**	0.85–0.96	**0.83**	0.68–0.91
Speech production	**0.85**	0.73–0.93	**0.90**	0.81–0.95
Self-esteem	**0.72**	0.52–0.86	**0.70**	0.49–0.85
Activity	**0.77**	0.59–0.88	**0.87**	0.75–0.93
Social interactions	**0.81**	0.66–0.90	**0.73**	0.53–0.86

**Abbreviation:** 95% CI, 95% confidence interval; CI, cochlear implant; ICC, intraclass correlation coefficient.

## Discussion

This study found that the DA-NCIQ has good internal consistency based on the values of the Cronbach alpha coefficients and good test–retest reliability based on the ICC values. The results support the use of this questionnaire as a reliable tool to measure the HRQoL in adult CI users.

Furthermore, this study found that participants with CIs had improved HRQoL compared to CI candidates, except for the advanced sound perception subdomain. Especially the basic sound perception, activity, and social interaction subdomains were found to be improved considerably in the post-CI group.

The most important or disabling aspect of a hearing impairment differs from individual to individual, also in a cross-cultural perspective. Using the NCIQ to detect changes in HRQoL after CI could make it easier to communicate the expected benefits of the surgery on an individual level.

Some differences are worth mentioning when comparing the findings in the present study with the original study 13 and the validation of NCIQ in respectively Italian and Spanish. 15 16 Hinderink et al. 13 and Sanchez-Cuadrado et al. 15 administered their versions of the NCIQ twice, once in the past tense to obtain retrospective information (pre-CI data) and once in the present tense to evaluate the current HRQoL (post-CI). The retrospective information (pre-CI data) was compared with a control group, which in Hinderink et al. 13 included patients on a waiting list for the implant. The comparison of the two groups revealed no significant differences. Ottaviani et al. 16 did a test–retest on the same group of participants, before and after they received the CI. They also included a control group consisting of postlingually deafened adults.

The pre-CI responses in the present study were not collected retrospectively, making this study more reliable than Hinderink et al. 13 and Sanchez-Cuadrado et al. 17

In the various versions of NCIQ, the scores for each subdomain were compared between the pre- and post-CI groups, including the present study, the original one by Hinderink et al., 15 and those conducted by Ottaviani et al. 18 and Sanchez-Cuadrado et al. 17 All of them revealed an overall improvement in scores for the post-CI group.

This difference identified between groups in all four studies underlines that the NCIQ is a relevant instrument to identify a change in HRQoL in adult postlingual deaf/hearing-impaired patients treated with CI.

In the present study, the highest score obtained for the post-CI group was in the activity subdomain (mean = 70.9). As a comparison, the highest score obtained for the same group was speech production for Hinderink et al. 13 (mean = 81.7), and advanced sound perception for both Ottaviani et al. 16 (mean = 82.9) and Sanchez-Cuadrado et al. 15 (mean = 82.8).

In the present study the post-CI group had the lowest scores for speech production (mean = 55.2), advanced sound perception for Hinderink et al. 13 (mean = 56.8), self-esteem for Ottaviani et al. 16 (mean = 63.6), and social interaction for Sanchez-Cuadrado et al. 15 (mean = 65.4). Furthermore, the pre-CI group had the highest scores for advanced sound perception (mean = 67.8), and the lowest for basic sound perception (mean = 44.8). As a comparison, speech production (mean = 59.8) and basic sound perception (mean = 3.2) showed the highest and lowest scores in the Hinderink et al. 13 study, respectively. As for Ottaviani et al., the social interaction (mean = 42.4) had the highest score, with the lowest being basic sound perception (mean = 31.5). 16 Finally, in Sanchez-Cuadrado et al., 15 the advanced sound perception (mean = 48.6) was the highest score, and basic sound perception (mean =19.3) had the lowest.

While there was no obvious connection between the highest and lowest scores in the post-CI group, the basic sound perception subdomain obtained the lowest scores in the pre-CI group in all four studies. Furthermore, the study of Hinderink et al. 13 showed considerably lower scores of the basic sound perception subdomain. 15 A possible explanation can be that their retrospective design is subject to recall bias. Another explanation could be that the participants had worse hearing before cochlear implantation in the Hinderink et al. 13 study compared to the present and other studies.

The set of 10 questions related to the basic sound perception subdomain assesses participants' ability to detect commonplace sounds such as car traffic, doorbells, toilet flushing, and similar auditory experiences. This underlines the significance of evaluating this form of sound perception as a crucial indicator of the advantages gained from the implant. These fundamental sound perception skills may not be fully captured by the conventional speech perception tests commonly employed as objective measures for evaluating the outcomes of CI.

Hinderink et al., 13 Ottaviani et al., 16 and Sanchez-Cuadrado et al. 15 all asked participants to complete an objective auditory performance test. Hinderink et al. 13 found poor correlations between all subdomains of the NCIQ and consonant-nucleus-consonant (CNC) words. This is also supported in a recent investigation where all subdomains of NCIQ had poor correlation with CNC words, the hearing in noise test, and AzBio sentence scores in quiet and in noise. 27 However, Ottaviani et al. 16 and Sanchez-Cuadrado et al. 15 both found a significant correlation between the results of word identification scores and the following subdomains: advanced sound perception, speech production, and self-esteem. Furthermore, Forli et al. 28 reported that CI is beneficial in regard to speech perception and HRQoL in patients over the age of 40 years.

Looking at the Cronbach alpha values, this study achieved satisfying values between 0.8 and 0.9 in all subdomains, with the exception of social interaction, in both groups (post-CI: α = 0.7; pre-CI: α = 0.79), and the speech production subdomain in the pre-CI group (α = 0.79).

Hinderink et al. 13 found Cronbach alpha values higher than 0.8 for all subdomains except for the speech production (α = 0.73) and the self-esteem (α = 0.75) subdomains.

Similarly, Ottaviani et al. 16 found Cronbach alpha values above 0.8 in all subdomains with the exception of the self-esteem subdomain, where the results in the different groups were pre-CI (α = 0.77), post-CI (α = 0.79), and control (α = 0.75).

Sanchez-Cuadrado et al. 15 found Cronbach alpha values higher than 0.7 in all subdomains, with the exception of social interaction (α = 0.65) in the control group, and speech production (α = 0.69) as well as self-esteem (α = 0.67) in the post-CI group.

Santos et al. 17 translated the NCIQ into Brazilian Portuguese and found Cronbach alpha values ranging from 0.72 to 0.92, with the subdomains self-esteem (α = 0.72), social interaction (α = 0.5), and basic sound perception (α = 0.76) having the lowest.

From a greater perspective, a tendency was detected towards the subdomains social interaction, self-esteem, and speech production, as they had the lowest Cronbach alpha values across several studies. Still, all the above-mentioned alpha values suggest that the internal consistency of the NCIQ is within a similar range, including when translated into other languages. It should, therefore, be possible to compare studies using NCIQ in different language versions.

It has been argued that the Cronbach alpha is not a good measure of internal consistency despite its widespread use in the literature. 29 However, as seen in this study, it is possible to compare alpha values between different language versions of the same questionnaire to get a picture of the different subdomains across different language versions. 29

The subdomains in the present study were found to have good or excellent reliability with ICC values ranging between 0.77 and 0.92, except for the following: self-esteem and social interaction in the post-CI group, as well as activity in the pre-CI group, which were found to have moderate reliability with ICC values, ranging between 0.7 and 0.77.

Hinderink et al. 13 found good reliability in the subdomains except for the Self-esteem subdomain with an ICC value of 0.62, which had moderate reliability. Ottaviani et al. 16 reported ICC values between 0.81 to 0.91, suggesting good reliability. However, since 95% confidence intervals were not provided by Hinderink et al. 13 and Ottaviani et al. 16 it is difficult to interpret the ICC values correctly, as the variation of the values is not known. Sanchez-Cuadrado et al. 15 did not perform a reliability analysis in their study.

During data collection in the present study, two new measurements of HRQoL in CI patients were published. McRackan et al. 30 had developed two new instruments: the cochlear implant quality of life (CIQOL)-35 profile and -10 global, from the CIQOL item bank. This item bank was based on 371 CI users' subjective identifications of HRQoL parameters. These authors argued that this patient involvement makes the questionnaires superior to the NCIQ, which was developed by experts. 30 A recent study also demonstrated that the CIQOL-35 profile and -10 global instruments were valid and reliable. This study also compared the NCIQ with the CIQOL-35. There was a reasonable correlation coefficient of 0.83 between the advanced sound perception domain of the NCIQ and the CIQOL-35's communication domain, as well as a correlation coefficient of 0.70 between the NCIQ activity limitation domain and the social interaction domain of the CIQOL-35. However, several constructs of the CIQOL-35 such as entertainment, environment, and listening effort, that were identified as important by CI users, are not covered in the NCIQ questionnaire. 27

### Strengths and Limitations

An answer rate of about 90% of all participants indicated that the chosen questionnaire was not too challenging to complete and was easily administered.

Some limitations in this study should be considered. First, the test–retest interval was only 2 weeks, and it is probable that some patients could have remembered their previous answers. Second, the sample size was limited, so additional studies of the DA-NCIQ are needed. Thirdly, the speech identification scores were not collected after the pre-CI group had undergone surgery. Therefore, a more relevant objective auditory performance test could have been selected to investigate the objective auditory benefit of the implant and compare it with the subjective domain scores of the DA-NCIQ. Furthermore, the CI usage time per day could have been a relevant benefit measurement to include in the study.

To investigate the benefits of CI, a comparison of NCIQ scores from before and after implantation was made. Further studies should consider comparing pre-CI scores with scores from the same group but obtained after surgery, for instance, 1-year postimplantation.

In the process of data analysis, certain questions, specifically questions 6 and 8, were frequently omitted from the dataset due to the considerable number of participants selecting the ‘not applicable’ response option. Both of these questions pertain to the social interaction domain. Notably, questions related to interactions with individuals who are deaf were excluded primarily due to the prevalence of the ‘not applicable’ response category.

Furthermore, it was observed that the Danish term employed for the translation of ‘hand gestures’ in question 15 (gestik) led to instances of confusion, as certain participants were unfamiliar with it. Consequently, it is advisable to review and reconsider the translation of this question in any forthcoming revisions.

## Conclusion

In research and practice, it is important to have a standardized, CI-specific HRQoL measurement, to enable predicting and assessing the benefits of implantation as well as comparing outcomes in different studies.

This study investigated the auditory performance of Danish CI users and candidates, as well as the internal consistency and test–retest reliability of the DA-NCIQ.

Even though additional research is required, especially focusing on the correlation between objective auditory performance tests and this questionnaire, the present study found that participants with CIs had an improved HRQoL related to all subdomains, except the advanced sound perception one, compared to their situation before CI.

Furthermore, the DA-NCIQ was found to have a good internal consistency with the Cronbach alpha coefficients between 0.7 and 0.91, and test–retest reliability with ICC values between 0.7 and 0.92, comparable to other language versions. Therefore, the results supported using this questionnaire as a reliable tool to measure the subjective benefits of CI in postlingually deafened/hearing-impaired adults.
