# Supplementary material for: Evaluation of the Nijmegen Cochlear Implant Questionnaire in Danish
Source: Int Arch Otorhinolaryngol. 2025 Jan 22;29(1):1–8. doi: 10.1055/s-0044-1788598 (PMC11753863; doi:10.1055/s-0044-1788598)
Supplement: Supplementary file 1 — Supplementary Material [file 10-1055-s-0044-1788598-s2022121455or.pdf]

Nijmegen Cochlear Implant Spørgeskema

Besvar venligst de følgende 60 spørgsmål vedrørende situationen med din høreteknik (fx høreapparat eller cochlear implantat). Brug svarmuligheden ”ikke relevant”, hvis ingen af svarmulighederne er relevante.

		Aldrig	Nogle gange	Ofte	For det meste	Altid	Ikke relevant
1	Kan du høre baggrundslyde (fx lyden af toiletskyl eller en støvsuger)?	☐	☐	☐	☐	☐	☐
2	Udgør din hørenedsættelse en stor hindring i din kontakt med personer med normal hørelse?	☐	☐	☐	☐	☐	☐
3	Kan du hviske, hvis du bliver nødt til det?	☐	☐	☐	☐	☐	☐
4	Føler du dig godt tilpas i andre selskab på trods af din hørenedsættelse?	☐	☐	☐	☐	☐	☐
5	Kan du føre en samtale i rolige omgivelser med én person (med eller uden mundaflæsning)?	☐	☐	☐	☐	☐	☐
6	Udgør din hørenedsættelse et stort problem i forbindelse med dit arbejde eller studier?	☐	☐	☐	☐	☐	☐
7	Kan du høre andres fodtrin derhjemme (fx hvis du går i gangen eller på trapperne)?	☐	☐	☐	☐	☐	☐
8	Udgør din hørenedsættelse et stort problem i kontakten med personer, der er døve?	☐	☐	☐	☐	☐	☐
9	Kan du råbe hvis du bliver nødt til det?	☐	☐	☐	☐	☐	☐
10	Generer det dig, at du er hørehæmmet?	☐	☐	☐	☐	☐	☐
11	Kan du føre en samtale med to eller flere personer i rolige omgivelser (med eller uden mundaflæsning)?	☐	☐	☐	☐	☐	☐
12	Er din hørenedsættelse et stort problem i trafikken?	☐	☐	☐	☐	☐	☐
13	Kan du høre, når din telefon eller dørklokke ringer?	☐	☐	☐	☐	☐	☐
14	Udgør din hørenedsættelse et stort problem, når du befinder dig i en gruppe af mennesker (fx fritidsaktiviteter, sport, ferie)?	☐	☐	☐	☐	☐	☐
15	Kan du gøre dig selv forståelig for personer du ikke kender uden brug af gestik?	☐	☐	☐	☐	☐	☐
16	Bliver du irriteret, hvis du ikke kan følge med i en samtale?	☐	☐	☐	☐	☐	☐
17	Når du er i en travl forretning, kan du så forstå ekspedienten?	☐	☐	☐	☐	☐	☐
18	Udgør din hørenedsættelse et stort problem ved fritidsaktiviteter?	☐	☐	☐	☐	☐	☐
19	Kan du høre (ikke føle), hvis hoveddøren smækker, mens du er travlt optaget derhjemme?	☐	☐	☐	☐	☐	☐
20	Er din hørenedsættelse et stort problem i kontakten med de personer, du bor sammen med (fx familie/partner)?	☐	☐	☐	☐	☐	☐
		Aldrig	Nogle gange	Ofte	For det meste	Altid	Ikke relevant
21	Kan du tilpasse din stemme til forskellige situationer (fx støjende omgivelser, rolige omgivelser)?	☐	☐	☐	☐	☐	☐
22	Undgår du at tale med personer du ikke kender?	☐	☐	☐	☐	☐	☐
23	Kan du nyde musik?	☐	☐	☐	☐	☐	☐
24	Udgør din hørenedsættelse et stort problem for dig i hjemmet?	☐	☐	☐	☐	☐	☐
25	Kan du høre biler der nærmer sig i trafikken?	☐	☐	☐	☐	☐	☐
26	Bliver du ladt alene, når du er i selskab med andre, på grund af din hørenedsættelse?	☐	☐	☐	☐	☐	☐
27	Kan personer du ikke kender høre på din stemme, at du er døv eller har en hørenedsættelse?	☐	☐	☐	☐	☐	☐
28	Beder du andre personer om at tale højere eller tydeligere, hvis de taler for lavt eller utydeligt?	☐	☐	☐	☐	☐	☐

(*Continued*)

 © 2025. The Author(s).

(*Continued*)

		Aldrig	Nogle gange	Ofte	For det meste	Altid	Ikke relevant
29	Kan du genkende visse melodier i musik?	☐	☐	☐	☐	☐	☐
30	Udgør din hørenedsættelse et stort problem, når du er på indkøb?	☐	☐	☐	☐	☐	☐
31	Kan du høre svage lyde (fx nøgler der falder, en mikrobølgeovn der bipper)?	☐	☐	☐	☐	☐	☐
32	Tager du steder hen hvor din hørenedsættelse kan udgøre en stor udfordring?	☐	☐	☐	☐	☐	☐
33	Kan du gøre dig forståelig for bekendte uden brug af gestik?	☐	☐	☐	☐	☐	☐
34	Føler du dig nervøs, når du taler med personer, du ikke kender?	☐	☐	☐	☐	☐	☐
35	Kan du genkende visse rytmer i musik?	☐	☐	☐	☐	☐	☐
36	Udgør din hørenedsættelse et stort problem, når du ser tv?	☐	☐	☐	☐	☐	☐
37	Kan du høre (ikke føle), når en person nærmer sig bagfra?	☐	☐	☐	☐	☐	☐
38	Udgør din hørenedsættelse en stor hindring i kontakten med personer i dit nabolag?	☐	☐	☐	☐	☐	☐
39	Hvor ofte er du generet af, at personer kan høre på din stemme/tale, at du har en hørenedsættelse?	☐	☐	☐	☐	☐	☐
40	Kan du forstå personer du ikke kender uden af mundaflæse?	☐	☐	☐	☐	☐	☐
41	Udgør din hørenedsættelse et stort problem til fester (fx fødselsdage)?	☐	☐	☐	☐	☐	☐
42	Kan du høre (ikke nødvendigvis forstå) personer, der taler i radioen?	☐	☐	☐	☐	☐	☐
43	Udgør din hørenedsættelse et stort problem, når du er sammen med venner?	☐	☐	☐	☐	☐	☐
44	Kan du nemt skabe kontakt med andre på trods af din hørenedsættelse?	☐	☐	☐	☐	☐	☐
		Aldrig	Nogle gange	Ofte	For det meste	Altid	Ikke relevant
45	Kan du høre forskel på en mandestemme, en kvindestemme og en barnestemme?	☐	☐	☐	☐	☐	☐
46	Udgør din hørenedsættelse et stort problem i forbindelse med formelle anliggender (fx forsikring, advokatbistand, kommunen)?	☐	☐	☐	☐	☐	☐
47	Kan du høre, når nogen kalder på dig?	☐	☐	☐	☐	☐	☐
48	Udgør din hørenedsættelse et stort problem i din kontakt med familiemedlemmer?	☐	☐	☐	☐	☐	☐
49	Er der situationer, hvor du ville føle dig gladere, hvis du ikke havde en hørenedsættelse?	☐	☐	☐	☐	☐	☐
50	Synes du, det er trættende at lytte (med eller uden mundaflæsning)?	☐	☐	☐	☐	☐	☐
51	Udgør din hørenedsættelse et stort problem, når du går ud eller tager på tur?	☐	☐	☐	☐	☐	☐
52	Kan du høre stemmer fra andre rum (fx børn der leger, en baby der græder)?	☐	☐	☐	☐	☐	☐
53	Når du befinder dig i en gruppe, føler du så, at din hørenedsættelse bevirker, at andre ikke tager dig alvorligt?	☐	☐	☐	☐	☐	☐
54	Påvirker din hørnedsættelse din selvtillid negativt?	☐	☐	☐	☐	☐	☐
55	Afholder din hørenedsættelse dig fra at forsvare dig selv (fx på arbejde, i forhold)?	☐	☐	☐	☐	☐	☐

 © 2025. The Author(s).

Bemærk at svarkategorierne for de 5 sidste spørgsmål er anderledes:

		Slet ikke	I ringe grad	I nogen grad	I høj grad	I meget høj grad	Ikke relevant
**56**	Er du i stand til at få din stemme til at lyde vred, venlig eller ked af det?	☐	☐	☐	☐	☐	☐
**57**	Kan du styre tonehøjden af din stemme (høj, lav)?	☐	☐	☐	☐	☐	☐
**58**	Kan du styre lydstyrken af din stemme?	☐	☐	☐	☐	☐	☐
**59**	Kan du få din stemme til at lyde ”naturlig” (så den ikke lyder døvepræget)?	☐	☐	☐	☐	☐	☐
**60**	Kan du gennemføre en enkel telefonsamtale?	☐	☐	☐	☐	☐	☐

 © 2025. The Author(s).
